# Probabilistic walking models using built environment and sociodemographic predictors

**DOI:** 10.1186/s12963-019-0186-8

**Published:** 2019-06-03

**Authors:** Anne Vernez Moudon, Ruizhu Huang, Orion T. Stewart, Hannah Cohen-Cline, Carolyn Noonan, Philip M. Hurvitz, Glen E. Duncan

**Affiliations:** 1 Architecture, Landscape Architecture, and Urban Design and Planning, University of Washington, 1107 NE 45th St, Suite 535, Box 354802, Seattle, WA 98195 USA; 2 Texas Advanced Computing Center, University of Texas at Austin, Austin, USA; 3 Present Address: Institute for Population Health Improvement, University of California, Davis, 4800 2nd Avenue, Suite 2600, Sacramento, CA 95817 USA; 4 Center for Outcomes Research and Education, Portland, OR 97213 USA; 5 Initiative for Research and Education to Advance Community Health (IREACH), Washington State University, Seattle, WA 98101 USA; 6 Department of Urban Design and Planning, University of Washington, Seattle, USA; 7 Elson S. Floyd College of Medicine, Department of Nutrition & Exercise Physiology, Washington State Twin Registry, Washington State University Health Sciences Spokane, Spokane, USA

**Keywords:** Physical activity, Active travel, Home neighborhood domains

## Abstract

**Background:**

Individual sociodemographic and home neighborhood built environment (BE) factors influence the probability of engaging in health-enhancing levels of walking or moderate-to-vigorous physical activity (MVPA). Methods are needed to parsimoniously model the associations.

**Methods:**

Participants included 2392 adults drawn from a community-based twin registry living in the Seattle region. Objective BE measures from four domains (regional context, neighborhood composition, destinations, transportation) were taken for neighborhood sizes of 833 and 1666 road network meters from home. Hosmer and Lemeshow’s methods served to fit logistic regression models of walking and MVPA outcomes using sociodemographic and BE predictors. Backward elimination identified variables included in final models, and comparison of receiver operating characteristic (ROC) curves determined model fit improvements.

**Results:**

Built environment variables associated with physical activity were reduced from 86 to 5 or fewer. Sociodemographic and BE variables from all four BE domains were associated with activity outcomes but differed by activity type and neighborhood size. For the study population, ROC comparisons indicated that adding BE variables to a base model of sociodemographic factors did not improve the ability to predict walking or MVPA.

**Conclusions:**

Using sociodemographic and built environment factors, the proposed approach can guide the estimation of activity prediction models for different activity types, neighborhood sizes, and discrete BE characteristics. Variables associated with walking and MVPA are population and neighborhood BE-specific.

**Electronic supplementary material:**

The online version of this article (10.1186/s12963-019-0186-8) contains supplementary material, which is available to authorized users.

## Background

Physical inactivity is a major public health concern. Two decades after developing programs and incentives to increase activity, the Centers for Disease Control and Prevention’s national surveillance data show that only about half of the US population reports engaging in at least 150 min of moderate-intensity physical activity per week [1], which is the level recommended for health [2]. More alarmingly, objective (meaning measured) data from a large representative sample show that the proportion of people who engage in recommended levels of activity is much smaller, at about 5% of the population [3].

Activity levels are thought to be affected by factors ranging from biology to policy (e.g., zoning laws). Research has investigated how physical activity, and particularly walking, is influenced not only at the individual level by demographic and social factors, as is common in public health, but also at the community and population levels, by the built environment (BE) and policies [4–6]. The BE is of interest to those working to increase physical activity because, unlike individual demographics and social factors, it can be directly modified to support activity, and its modifications can impact the entire populations [7–9].

“Walkability” is a catch-all term, which has been used to characterize activity-supportive BEs. Walkable environments are known to contain a transportation infrastructure that ensures short, direct, and safe trips to routine or recreational destinations while encouraging modes of travel other than automobiles [10, 11]. Routine destinations are shown to be associated with walking consist of transit stops, grocery stores, banks, coffee shops, and other retail outlets [12–14]. Recreational destinations associated with walking include parks, bodies of water, and facilities such as gyms or recreation centers [15, 16]. However, finding common links between environment and activity remains challenging because the relationship between walkability and actual activity levels, including walking, is complex. Across studies, variations in the conceptualization, measurement, and modeling of activity-friendly environments [17–19] impede the potential for generalization. Only a consistent treatment of environmental predictors and activity outcomes will help untangle their relationship for different populations in different settings [17, 20].

Methodological advantages may accrue from studies using individuals and their environment as units of analysis because they simplify the modeling process, facilitate the interpretation of results, and yield more statistical power [21]. Additionally, the increasing availability of detailed objective data on BE can help avert statistical errors due to the “modifiable areal unit problem,” which occurs when different spatial data are captured at different scales (e.g., counties and ZIP Codes) and/or in spatial units of different shapes (e.g., grids versus “natural” shapes) [22, 23]. As well, disaggregating these data facilitates targeting location-specific BE changes, which may be associated with increased physical activity [24, 25]. Yet with some exceptions [26, 27], few studies have combined both individual-level and disaggregated objective activity and BE data in the context of large populations living within large metropolitan regions.

This study presents an approach to estimate the probability that an individual will engage in physical activity at levels sufficient for health benefits. Using a large population sample in a large metropolitan area, it introduces a clear conceptual model of BE and a robust method to test the predictive ability of both sociodemographic factors and extensive objective measurements of BE characteristics on two outcomes of interest: amount of walking in the neighborhood and total moderate-to-vigorous physical activity (MVPA). The intent is to present a method to identify a concise set of variables that could eventually help construct a walking index combining measured behaviors with walkability indicators.

## Methods

### Participants

**Fig. 1:**
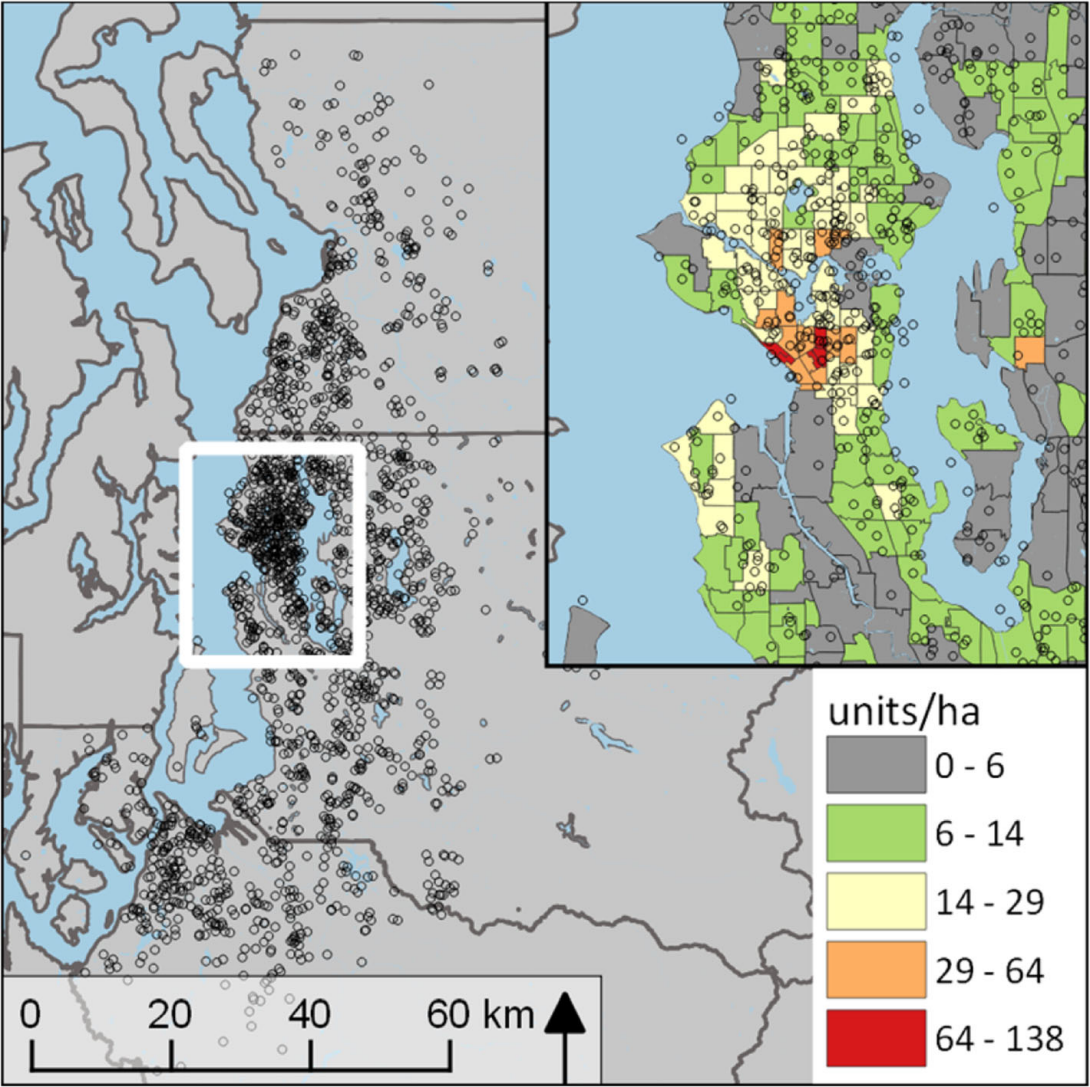
Map of the four Puget Sound region counties showing the residential locations of the 2497 participants in the study. The insert zooms into the City of Seattle against a background of residential unit densities by census tract (US Census ACS 2016). The exact participant XY locations have been jittered by a random value ± 1 km for purposes of de-identification (explaining why some participants’ locations are shown to be in Lake Washington)

Participants came from a community-based sample of adult twins assembled from the Washington State Department of Licensing records; a detailed description is available elsewhere [28]. The sample consisted of 2497 individual members of twin pairs living within the four-county Puget Sound region around Seattle, WA (population 3.3 million; 2600 km^2^ [1000 mi^2^]) (Fig. 1). Addresses and survey data, including items on sociodemographics, height, weight, general health, common medical conditions, and lifestyle behaviors, came from a Health & Wellbeing questionnaire administered between May 2010 and July 2012. The questionnaire followed an initial Twin Registry enrollment survey and was sent to 11,822 Registry members. The response rate was 74%, with only residents of the Puget Sound region selected for this study. Respondents provided written informed consent as a part of survey completion, and all study procedures were approved by the University of Washington institutional review board.

### Physical activity and sociodemographic measures

We assessed two distinct types of physical activity, MVPA and walking. MVPA came from two survey questions: (1) “Over the past 4 weeks, how many days during a typical week did you exercise vigorously for at least 20 minutes? Vigorous exercise causes heavy sweating or large increases in breathing or heart rate and includes such activities as running, lap swimming, aerobics classes, and fast bicycling” and (2) “Over the past 4 weeks, how many days during a typical week did you exercise moderately for at least 30 minutes? Moderate exercise causes only light sweating or light to moderate increases in breathing or heart rate and includes such activities as brisk walking, bicycling for pleasure, golf, and dancing.” The number of minutes per week of combined vigorous and moderate exercise was calculated as (20 min vigorous exercise × number of days) + (30 min moderate exercise × number of days). For the analyses, MVPA was dichotomized as < 150 and ≥ 150 min per week, the recommended threshold of physical activity [2, 29].

Walking was assessed with two questions: (1) “How many days during a typical week do you walk for recreation, exercise, to get to and from places, or for any other reason in your neighborhood?” and (2) “When you walk in your neighborhood, about how many minutes, on average, do you spend walking each time you walk?” Response options, in minutes, included < 15, 15, 30, 45, 60, 75, and > 90. The question was phrased so that respondents could include all types of walking, brisk or casual, as well as utilitarian or recreational, in their assessment. The number of minutes per week of walking was calculated as (number of days × average number of minutes walking each time). When participants responded “less than 15” or “90 or more” minutes, the calculation used 10 or 90, respectively, for the average number of minutes. For the analyses, walking was also dichotomized as < 150 and ≥ 150 min per week.

Sociodemographic variables were age, sex, race (White and non-White), education level (less than high school graduate, high school graduate or general equivalency diploma, some college, and Bachelor’s degree or higher), and total household income in the past year (< $20,000; $20,000–29,999; $30,000–39,999; $40,000–49,999; $50,000–59,999; $60,000–69,999; $70,000–79,999; and ≥ $80,000).

### BE variables

Built environment variables came from four domains that previous studies have associated with walking and physical activity in general: regional context, neighborhood composition, destinations, and transportation [17, 30, 31]. Regional context was defined in terms of terrain (i.e., slope), because the Puget Sound region is notably hilly, and living in Seattle vs. not, because Seattle is its largest, most densely developed city. Variables for neighborhood composition included the density of residential units and jobs, the presence of neighborhood commercial centers, and residential property values. Destinations were divided into subcategories: food sources (e.g., grocery stores, restaurants), retail and service (e.g., general merchandise, health centers), sports and fitness facilities (e.g., team or solo sports), open space (e.g., parks), and education facilities (e.g., schools). Transportation included both infrastructure (e.g., streets, intersections) and traffic conditions (e.g., vehicular traffic volumes, bus ridership). The list of BE variables and related measures is presented in Additional file 1.

Regional terrain slope came from the USGS National Elevation Dataset (NED 1/3 arc-second raster data, 2012). Neighborhood composition data came from assessor’s tax parcel data in the Puget Sound four counties: King (2010), Snohomish (2009), Pierce (2009), and Kitsap (2009). Employment data were developed by the Urban Form Laboratory (UFL) team [32]. Data on food sources and facilities for fitness, services, and retail came from the commercial vendor InfoUSA (2011) and were classified by the UFL [33]. Parks data were developed by the UFL (2012) based on county and local jurisdictional data [34]. School data came from the National Center for Education Statistics [35]. Streets and intersections were derived from OpenStreetMap data (https://www.openstreetmap.org/#map=5/51.500/-0.100; 2015). Traffic volumes and bike facilities came from the Puget Sound Regional Council (http://www.psrc.org/data/transportation/bikeped-data; 2006). Bus ridership (2010, 2011) came from the region’s transit agencies.

### BE measures

Two commonly used radii defined the home neighborhood [10, 30, 31, 36, 37]. The immediate neighborhood lay 833 m (0.5 mi) from the home parcel, corresponding to a 10-min walking distance, and 1666 m (1 mi) defined the extended neighborhood within a 20-min walk. “Sausage” network buffers captured areas of exposure [36]. The buffers are created by first identifying street segments traversable within 833 m and 1666 m of each respondent’s home parcel, then by delineating areas within 100 m of street center lines, and by filling in any gaps inside the buffer.

Measures taken for each BE variable included count (e.g., of residential units, individual destinations, bus riders), length (e.g., of streets), area (e.g., of parks), density (e.g., gross density of residential units in the neighborhood, net density of residential units in the residential parcels of the neighborhood), and shortest route distance from participants’ homes. There were 86 initial BE measures. The regional context domain had 2 measures of 2 variables; the neighborhood composition domain had 13 measures of 5 variables; the destinations domain had 56 measures of 41 variables; and the transportation domain had 15 measures of 5 variables (Additional file 1). Based on OpenStreetMap, network buffer and distance measures were calculated in PostgreSQL 9.3.8 and PostGIS 2.1.3 (PostGIS Development Group, 2008) using programmatic wrappers in R 2.15.3.

### Geocoding

Twins’ home addresses and food establishments, fitness and sport facilities, general services, and retail businesses from the InfoUSA destinations data (see Additional file 1 for details) were geocoded in ArcGIS 10.2.1 (Redlands, CA) using StreetMapUSA Premium (http://www.esri.com/data/streetmap; 2014). For twins’ addresses, 60% were matched to a building rooftop point using a match score of 100, 20% were matched with street address interpolation, and 20% were matched manually. For InfoUSA destinations, a 60 minimum geocoding match score required manual geocoding of 36% of the food destinations (*n* = 8293), 33% of the fitness destinations (*n* = 1550), and 37% of the retail destinations (*n* = 7534). Fewer than 3% remained unmatched.

### Analyses

Descriptive statistics were computed as mean, standard deviation, and range or percent. Prediction models used MVPA and walking as two separate outcomes, and BE variables within the immediate neighborhood (833 m) and within the extended neighborhood (1666 m) as predictors. All four models included sociodemographic variables as predictors. All analyses treated twins as singletons, controlling where needed for correlations within twin pairs.

Modeling entailed four steps. First, to reduce the number of BE variables, the initial 86 measures were correlated with the two outcome variables in the two neighborhood sizes using Pearson correlation coefficients. Correlations were small, and the top 25 measures (correlation coefficient > 0.09) were retained for each one of the four models. Variables were then tested for multicollinearity, and only one of the highly correlated variables (> 0.8) was kept for inclusion in the models. The final number of BE variables entered in the models ranged from 9 to 16 depending on the model.

Second, we assessed the most appropriate form for independent variables by comparing continuous and categorical forms using the Hosmer and Lemeshow goodness-of-fit test [38]. Ultimately, we found that alternative forms did not improve predictive ability and we kept the sociodemographic and BE variables in continuous form for simplicity. The exceptions were the BE variables measuring the distance from participants’ homes to nearest BE feature (e.g., youth or solo sports facilities), which needed to be categorized in order to retain the large portion of the sample (25–37%) that had distances larger than the 1666 m extended neighborhood. The categorized distance variables were modeled in continuous form.

The data were divided into training and validation datasets, to develop the prediction models and to determine model fit avoiding artificially high estimates of model fit, respectively. Two thirds of the twin pairs were randomly selected for the training dataset and one third for the validation dataset. The use of twin pairs, not individuals, insured that the training and validation datasets were independent from each other.

Third, a backward elimination approach established the best subset of independent variables to include in each one of the four models and to constitute what we called “index variables.” A first logistic regression model included all sociodemographic and BE variables. The variable with the largest *P* value was then removed and the model re-fit iteratively, until all remaining predictors had *P* values ≤ 0.20 (the traditional 0.05 threshold might reduce the power to find appropriate predictors). Age and sex were forced into the models along with education or income or both, as these four factors are widely associated with physical activity and walking [17]. If neither education nor income had *P* ≤ 0.20, then the variable with the lowest *P* value was included. Clustered robust standard errors served to properly inflate variance estimates due to the correlation within twin pair.

Fourth, we assessed the best subset model using the goodness-of-fit test and receiver operating characteristic (ROC) curves in the validation dataset. Area under the ROC curve or the C-statistic (0.0–1.0) was used, where 0.5 represented no predictive ability beyond random chance and 1.0 indicated strong predictive ability. The ROC curve from the best subset model was compared to a model including only sociodemographic variables to determine if the best subset model differed significantly in predictive ability. All analyses used Stata/SE 12.1 (College Station, TX).

## Results

Out of 2497 participants, 105 (4%) were excluded due to missing data for sociodemographic, MVPA, or walking variables. Of the 2392 remaining participants, more than 60% were female, almost 90% were White, and the average age was under 40. About 50% had a college degree, and more than 60% had annual household incomes above $50,000.

Table 1 shows sociodemographic factors and levels of physical activity stratified by the 150 min per week cut point. Only 39% of participants reported at least 150 min of MVPA per week, and only 24% reported at least 150 min of walking per week. For those reporting less than 150 min per week of MVPA, the mean was 61.8 min, while it was 209.8 min for the more active group of participants (overall mean of 120 min). For walking in the neighborhood, the reported mean minutes per week was 93 min, with 47.4 and 238.9 min reported by the two groups of participants, respectively.

**Table 1:**
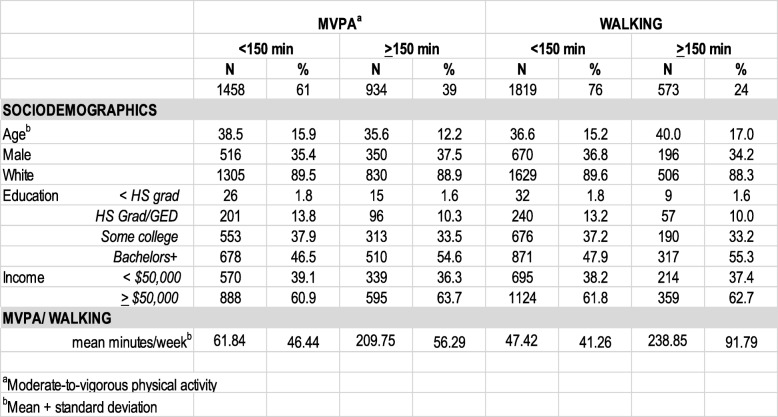
Descriptive statistics of the sample by level of physical activity and walking

^a^Moderate-to-vigorous physical activity

^b^Mean + standard deviation

Table 2 shows the sociodemographic and BE predictor variables selected for inclusion in the models. Of the four sociodemographic variables, age, sex, and education remained in all final models, but race was eliminated, as was income in the walking models.

**Table 2:**
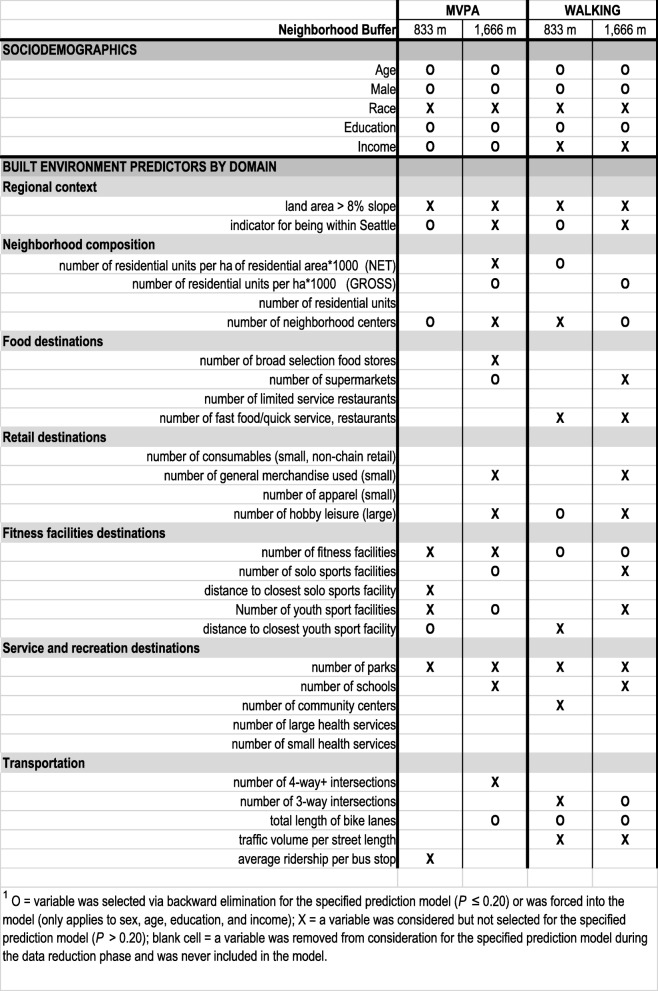
Sociodemographic and domain-specific built environment variable selection by activity type and neighborhood size

O = variable was selected via backward elimination for the specified prediction model (*P* ≤ 0.20) or was forced into the model (only applies to sex, age, education, and income); X = a variable was considered but not selected for the specified prediction model (*P* > 0.20); blank cell = a variable was removed from consideration for the specified prediction model during the data reduction phase and was never included in the model

After testing for multicollinearity, BE variables included in the final models came from all BE domains. For MVPA, 3 out of 9 and 5 out of 16 BE variables remained in the models of the immediate and extended neighborhoods, respectively. For the immediate neighborhood, being in Seattle, the number of neighborhood centers (containing at least a retail outlet, a grocery store, and a traditional restaurant) and distance to the nearest youth facility were associated with meeting the 150 min per week MVPA threshold. For the extended neighborhood, gross residential density, the number of supermarkets, solo sport (e.g., boxing, water sports, bicycle rental, golf, tennis, and climbing, as different from team sports, such as football, basketball, baseball, and soccer), and youth facilities remained in the model, as well as the total length of bike lanes.

For walking, 5 each out of 15 and 13 BE variables remained in the immediate and extended neighborhood models, respectively. Both walking models included residential density (net and gross), the number of fitness facilities, and the total length of bike lanes. The 833-m neighborhood walking model also retained being in Seattle and the number of hobby-leisure facilities, while in the 1666-m model, the number of neighborhood centers and the number of 3-way street intersections remained as predictors of walking at least 150 min per week.

Neither sociodemographic nor BE variables remaining as index variables in the models had strong predictive ability for MVPA or walking (Table 3). Age and sex variables had different associations with walking and MVPA. Age was negatively related to engaging in sufficient MVPA, but positively associated with walking at least 150 min per week. Males were more likely to engage in MVPA but less likely to walk than their female counterparts. Participants with higher incomes were more likely to reach the recommended MVPA level. In terms of neighborhood BE characteristics, participants who reached the 150-min activity threshold were more likely to be located in Seattle, to live in denser neighborhoods with more neighborhood centers and destinations than those who did not reach the health-benefit threshold.

**Table 3:** .

Variable	MVPA^a^						WALKING					
	833 m			1666 m			833 m			1666 m		
	OR	95% CI	*P*-value	OR	95% CI	*P*-value	OR	95% CI	*P*-value	OR	95% CI	*P*-value
Sociodemographic												
Age	0.984	0.974, 0.995	0.004	0.984	0.973, 0.994	0.003	1.017	1.006, 1.029	0.003	1.018	1.006, 1.029	0.003
Male	1.379	0.994, 1.914	0.054	1.384	0.993, 1.927	0.055	0.845	0.593, 1.203	0.35	0.831	0.583, 1.184	0.305
Education	1.188	0.954, 1.477	0.124	1.191	0.957, 1.484	0.117	1.202	0.931, 1.551	0.157	1.229	0.959, 1.576	0.104
Household income	1.087	1.023, 1.155	0.007	1.093	1.028, 1.162	0.004						
Regional location												
Seattle	1.534	0.961, 2.447	0.073				1.518	0.948, 2.430	0.082			
Neighborhood composition												
Res units/ha (GROSS)				1.32	0.976, 1.786	0.071				1.477	0.942, 2.316	0.089
Res units/ha of res area (NET)							1.024	0.996, 1.054	0.098			
Neighborhood centers (count)	0.794	0.600, 1.512	0.107							1.181	0.979, 1.423	0.082
Destinations												
Supermarkets (count)				0.852	0.715, 1.014	0.071						
Hobby leisure (count)							2.762	0.890, 8.568	0.079			
Fitness facilities (count)							0.916	0.839, 0.999	0.047	0.914	0.860, 0.970	0.003
Solo sports facilities (count)				1.279	1.080, 1.514	0.004						
Youth sport facilities (count)				1.111	0.980, 1.257	0.098						
Youth sport facility (distance)	0.941	0.862, 1.027	0.175									
Transportation												
3-way intersections (count)										1.001	1.000, 1.003	0.114
Bike lanes (total length)				1	1.000, 1.000	0.019	1	1.000, 1.000	0.128	1	1.000, 1.000	0.069
_cons	0.38	0.155, 0.931	0.034	0.269	0.119,0.610	0.002	0.069	0.026, 0.184	<0.001	0.041	0.014, 0.120	<0.001

^a^Moderate-to-vigorous physical activity

**Fig. 2:**
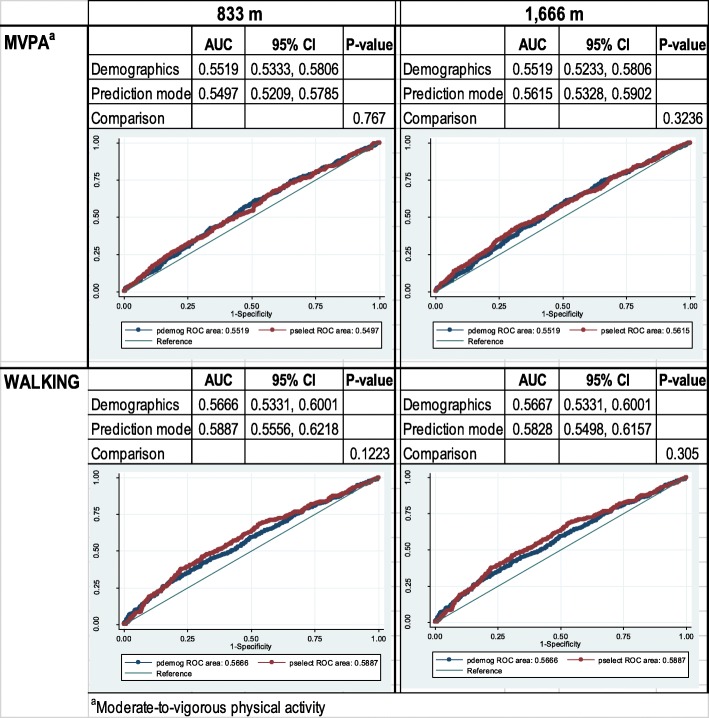
Comparison of receiver operating characteristic (ROC) curves for sociodemographic models (pdemog) versus best models including BE variables (pselect)

Comparing the ROC curve from the best subset models to that from models including only sociodemographic variables indicated that the addition of BE variables did not significantly improve predictive ability (Fig. 2). The area under the curve (AUC) of the walking models was 0.59 (95% CI 0.56, 0.62) and 0.58 (95% CI 0.55, 0.62) for the immediate and the extended neighborhood, respectively. It was lower, at 0.55 (95% CI 0.52, 0.58) and 0.56 (95% CI 0.53, 0.59), for the 833-m and the 1666-m MVPA models, respectively.

## Discussion

The proposed method addressed the challenges of assessing the influence of home neighborhood BE characteristics on the probability of being active. It used a clear conceptual construct of the home neighborhood BE and a comprehensive and consistent treatment of BE characteristics that can be replicated in other studies. The method demonstrated how to systematically reduce the large number of variables and measures necessary to characterize the BE in the modeling process. The final number of BE variables was down to a manageable maximum of 5 per model. Additionally, ROC analyses helped to economically compare the predictive ability of sociodemographic and home neighborhood BE characteristics in assessing the likelihood of an individual meeting the recommended level of physical activity or walking.

In this study, BE characteristics did not improve models predicting walking or MVPA at levels sufficient to benefit the health of adults, implying that for this population, higher neighborhood walkability might not lead to more total MVPA or more neighborhood walking [39, 40]. However, as different BE variables were retained for models predicting MVPA versus walking within the immediate and the extended neighborhood, results suggested that BE effects may vary by activity type and neighborhood size. Furthermore, the final BE index variables came from all of the proposed BE domains, confirming that these domains identify distinct aspects of BE walkability [17, 31]. Neighborhood composition (measured as density and mixed-use development) remained in all of the models. All models also contained at least one variable in the neighborhood destination domain, which was related to food, general retail, or sport activities, but none retained service and recreation destinations. Transportation domain variables remained in the two walking models and the 1666-m MVPA model. Regional location (measured as being within the City of Seattle) remained in the walking models. Of note, the index variables measured the effect of discrete attributes of the BE, thus potentially guiding interventions targeting changes to the BE that might lead to increased physical activity [21, 24, 25]. In contrast, models using factor analyses or other aggregation techniques to capture the BE cannot provide the information necessary to directly connect analytic results with intervention strategies or policy development.

Regarding the impact of sociodemographic characteristics, age, income, and education had the expected association with activity, in line with other studies [41]. The small proportion of participants reporting at least 150 min of MVPA (39.05%) was more than that of the US adult population as determined using objective measures [3], but less than that the proportion determined using national surveillance instruments [1]. Although this finding lent some support for the utility of our MVPA variable, it was unexpected given the relatively young age of the sample and considering the likelihood that participants over-reported their physical activity via self-report [29]. Also, the proportion of our sample reporting at least 150 min of walking per week was small (less than 24%) [42].

The study limitations included the use of survey-based outcomes, which could lead to over-reporting physical activity. However, in an ongoing study of twins who wore accelerometers and GPS devices over a 2-week period, subjectively measured MVPA of 283 twins correlated significantly with objectively measured MVPA (*r* = 0.47, *P <* 0.001) (Additional file 2). The correlation for walking was *r* = 0.44, *P* < 0.001. Further, the survey data covered total MVPA but only neighborhood walking, thus disallowing inferences on the influence of the home BE on either neighborhood MVPA or total walking [43]. Regarding the location of activity, we tested the effect of different neighborhood sizes in the probability of engaging in activity, but could not assess the potential impact of temporally different neighborhood exposures between participants [44]. The cross-sectional study design limits the ability to infer causation between the BE and active behaviors. Finally, lack of data on participant perceptions of their home neighborhood precluded estimating the effects of subjective neighborhood assessment on activity, potentially biasing the findings [45].

## Conclusions

The study introduced a clear approach to estimate the influence of sociodemographic and discrete attributes of the home neighborhood BE on an individual’s probability of being active or walking sufficiently to benefit health. It presented a method to identify BE variables of the immediate and extended home neighborhoods likely to be associated with activity.

For the study population, including BE variables in the models did not improve the ability to predict the likelihood of reaching recommended health-sustaining levels of walking or physical activity compared to using sociodemographic variables only. This does not mean that these relationships do not exist. Perhaps, different methods and objective data that would address possible measurement error in the outcome variables would yield different results. Further analyses of possible non-linear associations between BE and behavior outcome variables might also change the results. However, sociodemographic and BE variables associated with neighborhood walking at least 150 min per week differed from those associated with MVPA of the same duration, suggesting that different factors may be involved in the engagement of walking or MVPA. Sociodemographic and BE influences also varied by the assumed size of the home neighborhood. Final BE variables came from all four proposed BE domains, which should be considered in analyses of BE influences on activity in general.

## Additional files

Additional file 1: Built environment variables and measures by domain. The file contains a table of the 86 built environment variables and related measures included in the analyses. Variables are classified into the four main domains of the proposed BE conceptual construct, with subdomains identified when needed to further clarify the construct. (PDF 132 kb)

Additional file 2: Comparing measured with self-report MVPA and walking data using a sub-sample of 283 twins who wore accelerometers and GPS devices over a 2-week period. The file contains supplemental information on correlations between measured and self-report MVPA and walking. Also provided is a within-person comparison of measured and self-report walking data using a 150- and 100-min per week threshold of recommended activity. (PDF 138 kb)

## Abbreviations

BE: Built environment
MVPA: Moderate and vigorous physical activity
PA: Physical activity
ROC: Receiver operating characteristic
UFL: Urban Form Laboratory at the University of Washington
USGS NED: United States Geological Survey National Elevation Dataset
WA: Washington State
