# Supplementary material for: Probabilistic walking models using built environment and sociodemographic predictors
Source: Popul Health Metr. 2019 Jun 3;17:7. doi: 10.1186/s12963-019-0186-8 (PMC6547573; doi:10.1186/s12963-019-0186-8)
Supplement: Supplementary file 1 — Built environment variables and measures by domain. The file contains a table of the 86 built environment variables and related measures included in the analyses. Variables are classified into the four main domains of the proposed BE conceptual construct, with subdomains identified when needed to further clarify the construct. (PDF 132 kb) [file 12963_2019_186_MOESM1_ESM.pdf]

Additional file 1: Built environment variables and measures by domain

The following is a complete list of the BE variables included in the analyses. Variables are classified into the four main domains of the proposed BE conceptual construct, with subdomains identified when needed to further clarify the construct. Variable names and measures are also presented, totaling 86.

	DOMAIN	SUBDOMAIN	NAME	MEASURE	
1	**REGIONAL CONTEXT**		In or out of Seattle	In Seattle vs out	In: 1; Out: 0
2			Slope	area	Land area > 8% slope
3	**NEIGHBORHOOD COMPOSITION**	Residential units	Res density	count	Total number of residential units within buffer
4			Res density	gross density	Total number of residential units within buffer / buffer area
5			Res density	net density	Total number of residential units within buffer / residential parcel area within buffer
6		Jobs	Employment density	count	Total number of employees within buffer
7			Employment density	density	Total number of employees from major employers within buffer / buffer area
8		Housing type	Single-family (SF) number	count	Total number of SF units within buffer
9			Share of SF units	ratio	Total number of single family units within buffer / total number of residential units *100
10			Multifamily (MF) number	count	Total number of MF units within buffer
11			SF area	area	Total area of single family parcels within buffer
12			Share of SF area	ratio	Total area of single family parcels within buffer / buffer area *100
13			MF area	area	Total area of multi-family parcels within buffer
14		Neighborhood commercial center	Neighborhood center	count	Total number of neighborhood center (a polygon that contains at least 1 grocery, 1 traditional restaurant and 1 retail
15		Property value	Residential property value	Average residential property value per residential unit	Total residential property value within buffer / total number of units within buffer
16	**DESTINATIONS**	Food	Broad selection food stores	count	Total number of broad selection food stores within buffer

		establishments			
17				Network distance	Shortest distance to the closest of broad selection food store
18			Limited selection food stores	count	Total number of limited selection food stores within buffer
19				Network distance	Shortest distance to the closest of limited selection food store
20			Supermarket	count	Total number of supermarkets within buffer
21				Network distance	Shortest distance to the closest supermarket
22			Grocery store	count	Total number of grocery stores within buffer
23				Network distance	Shortest distance to the closest of grocery stores
24			Convenience store plus drug/food combos	count	Total number of convenience and drug/food stores within buffer
25				Network distance	Shortest distance to the closest of convenience and drug/food store
26			Full service restaurants	count	Total number of full service within buffer
27				Network distance	Shortest distance to the closest of full service
28			Limited service restaurants	count	Total number of limited service restaurants within buffer
29				Network distance	Shortest distance to the closest of limited service restaurants
30			Fast food plus quick service restaurants	count	Total number of fast food+quick service restaurants within buffer
31				Network distance	Shortest distance to the closest of fast food+quick service restaurant
32		Fitness facilities	Community center with PA	count	Total number of community centers with PA within buffer
33				Network distance	Shortest distance to the closest of community center with PA
34			Fitness facility	count	Total number of fitness facilities with PA within buffer
35				Network distance	Shortest distance to the closest of fitness facility
36			Leisure	count	Total number of leisure within buffer
37				Network distance	Shortest distance to the closest of leisure
38			Youth	count	Total number of youth within buffer
39				Network distance	Shortest distance to the closest of youth
40			Team sports	count	Total number of team sports within buffer
41				Network distance	Shortest distance to the closest of team sports

42			Solo sports	count	Total number of solo sports within buffer
43				Network distance	Shortest distance to the closest of solo sports
44		Retail and service establishments	General merchandise new small	count	Total number of general merchandise new small within buffer
45			General merchandise new medium	count	Total number of general merchandise new medium within buffer
46			General merchandise new large	count	Total number of general merchandise new large within buffer
47			General merchandise new all	count	Total number of general merchandise new within buffer
48			General merchandise used small	count	Total number of general merchandise used small within buffer
49			General merchandise used medium	count	Total number of general merchandise used medium within buffer
50			General merchandise used large	count	Total number of general merchandise used large within buffer
51			General merchandise used all	count	Total number of general merchandise used within buffer
52			Health small	count	Total number of health small within buffer
53			Health medium	count	Total number of health medium within buffer
54			Health large	count	Total number of health large within buffer
55			Health all	count	Total number of health within buffer
56			Hobby leisure small	count	Total number of hobby leisure small within buffer
57			Hobby leisure medium	count	Total number of hobby leisure medium within buffer
58			Hobby leisure large	count	Total number of hobby leisure large within buffer
59			Hobby leisure all	count	Total number of hobby leisure within buffer
60			Consumables small	count	Total number of consumables small within buffer
61			Consumables medium	count	Total number of consumables medium within buffer
62			Consumables large	count	Total number of consumables large within buffer
63			Consumables all	count	Total number of consumables within buffer
64			Apparel small	count	Total number of apparel small within buffer
65			Apparel medium	count	Total number of apparel medium within buffer
66			Apparel large	count	Total number of apparel large within buffer
67			Apparel all	count	Total number of apparel within buffer
68		Open space	Park	count	Total number of parks within buffer
69			Park	area	Total area of park within buffer

70		Education uses	School	count	Total number of schools within buffer
71			Campus	area	Total area of schools within buffer
72	**TRANSPORTATION INFRASTRUCTURE**	Street	Street density all streets	linear meter	Total length of streets within buffer
73			Streets < 40mph	linear meter	Total length of streets<40mph within buffer
74		Intersection	Intersection density (1 way)	count	Total number of 1 way street intersections within buffer
75			Intersection density (2 way)	count	Total number of 3 way street intersections within buffer
76			Intersection density (4 way)	count	Total number of 4 way+ street intersections within buffer
77		Bike lane	Bike lane all types	linear meter	Total length of bike lanes of all types within buffer
78			Bike lane separated	linear meter	Total length of separated bike lanes within buffer
79			Bike lane striped	linear meter	Total length of striped bike lanes within buffer
80			Shortest distance to separated bike lane	linear meter	Shortest distance to a separated bike lane
81			Shortest distance to striped bike lane	linear meter	Shortest distance to a striped bike lane
82	**TRAFFIC CONDITIONS**	Traffic volume	Vehicular volumes on all streets	Average Annual Daily Traffic (AADT)_all streets	Total traffic volume / total street length within buffer
83			Vehicular volumes on streets <40mph	Average Annual Daily Traffic (AADT)_street <40mph	Total traffic volume on streets <40mph /total length of street < 40mph within buffer
84			Shortest to arterials	Airline distance to arterial >5,000 volume	Shortest distance to arterial with greater than 5,000 vehicle volume
85		Bus ridership	Bus ridership	count	Total number of bus Boardings and Alightings within buffer
86			Average ridership per bus stop	Count per stop	Total number of bus Boardings and Alightings within buffer / number of bus stops within buffer
