# Supplementary material for: Probabilistic walking models using built environment and sociodemographic predictors
Source: Popul Health Metr. 2019 Jun 3;17:7. doi: 10.1186/s12963-019-0186-8 (PMC6547573; doi:10.1186/s12963-019-0186-8)
Supplement: Supplementary file 2 — Comparing measured with self-report MVPA and walking data using a sub-sample of 283 twins who wore accelerometers and GPS devices over a 2-week period. The file contains supplemental information on correlations between measured and self-report MVPA and walking. Also provided is a within-person comparison of measured and self-report walking data using a 150- and 100-min per week threshold of recommended activity. (PDF 138 kb) [file 12963_2019_186_MOESM2_ESM.pdf]

Additional file 2: Comparing measured with self-reported MVPA and walking data using a sub-sample of 283 twins who wore accelerometers and GPS devices over a two-week period

Supplemental information is provided on correlations between self-report and measured MVPA and walking. Also provided is a within-person comparison of self-report and measured walking data using the 150-minute per week threshold of recommended activity.

1. **Correlation between measured MVPA and self-report MVPA**

Used bout minutes per week based on actual number of weeks with collected data and self-report MVPA questions (in past 4 weeks, how many days in a typical week with 20 minutes vigorous/30 minutes moderate activity?).

Paired Samples Statistics

		Mean	N	Std. Deviation	Std. Error Mean
Pair 1	meas_mvpa	111.146	283	115.6454	6.8744
	sr_mvpa	119.72	283	88.285	5.248

Paired Samples Correlations

		N	Correlation	Sig.
Pair 1	meas_mvpa & sr_mvpa	283	.472	.000

Measured MVPA and self-report MVPA are moderately correlated. Mean difference between measured MVPA and self-report MVPA is -8.57 (SD 107.3), but this difference is not significant.

a. Does this correlation vary by age?

Correlations

			sr_mvpa
**38 and under**	**meas_mvpa**	**Pearson Correlation**	**.617**
		**Sig. (2-tailed)**	**.000**
		**N**	**95**
39-50	meas_mvpa	Pearson Correlation	.366
		Sig. (2-tailed)	.000
		N	94
51 and older	meas_mvpa	Pearson Correlation	.435
		Sig. (2-tailed)	.000
		N	94

Yes, correlation is highest in twins under 38.

b. *Does this correlation vary by sex?*

Correlations

			sr_mvpa
**Female**	**meas_mvpa**	**Pearson Correlation**	**.565**
		**Sig. (2-tailed)**	**.000**
		**N**	**203**
Male	meas_mvpa	Pearson Correlation	.271
		Sig. (2-tailed)	.015
		N	80

Yes, higher in female twins (p <.01)

2. Correlation between measured neighborhood walking and self-report neighborhood walking

Used walking minutes per week based on actual number of weeks with collected data and self-report walking questions (how many days walked in a typical week, how many minutes).

Paired Samples Statistics

		Mean	N	Std. Deviation	Std. Error Mean
Pair 1	sr_walk	91.41	281	95.359	5.689
	meas_walk	66.196	281	93.9141	5.6024

Paired Samples Correlations

		N	Correlation	Sig.
Pair 1	sr_walk & meas_walk	281	.436	.000

Like MVPA, measured and self-report walking are moderately correlated. Unlike MVPA, the mean difference between self-report and measured walking is significant (25.2, SD 100.5).

a. *Does this correlation vary by age?*

Correlations

agegroup			sr_walk
38 and under	meas_walk	Pearson Correlation	.262
		Sig. (2-tailed)	.012
		N	92
39-50	meas_walk	Pearson Correlation	.327
		Sig. (2-tailed)	.001
		N	95
**51 and older**	**meas_walk**	**Pearson Correlation**	**.646**
		**Sig. (2-tailed)**	**.000**
		**N**	**94**

Yes, higher correlation is in the oldest twins

b. Does this correlation vary by sex?

Correlations

male			sr_walk
Female	meas_walk	Pearson Correlation	.410
		Sig. (2-tailed)	.000
		N	201
Male	meas_walk	Pearson Correlation	.573
		Sig. (2-tailed)	.000
		N	80

Yes, but the difference is not significant

3. Comparing self-report with measured walking, 150-minute cutoff

As seen in the following table, 16.9% of twins reported walking more than 150 minutes in a typical week but measured less than 150 minutes per week during data collection, while 4.6% reported walking less than 150 minutes in a typical week but measured more than 150 minutes of walking per week. Using different walking cutoffs did not improve the number of twins who were "misclassified."

Self-reported walking * Measured walking Crosstabulation

			Measured walking		
			Less than 150	>= 150 minutes	Total
Self-reported walking	Less than 150	Count	199	13	212
		% of Total	70.1%	4.6%	74.6%
	>= 150 minutes	Count	48	24	72
		% of Total	16.9%	8.5%	25.4%
Total		Count	247	37	284
		% of Total	87.0%	13.0%	100.0%
